# Neuroprotection, Growth Factors and BDNF-TrkB Signalling in Retinal Degeneration

**DOI:** 10.3390/ijms17091584

**Published:** 2016-09-20

**Authors:** Atsuko Kimura, Kazuhiko Namekata, Xiaoli Guo, Chikako Harada, Takayuki Harada

**Affiliations:** Visual Research Project, Tokyo Metropolitan Institute of Medical Science, 2-1-6 Kamikitazawa, Setagaya-ku, Tokyo 156-8506, Japan; kimura-at@igakuken.or.jp (A.K.); guo-xl@igakuken.or.jp (X.G.); harada-ck@igakuken.or.jp (C.H.); harada-tk@igakuken.or.jp (T.H.)

**Keywords:** BDNF, TrkB, retinal degeneration, glaucoma, neuroprotection, valproic acid

## Abstract

Neurotrophic factors play key roles in the development and survival of neurons. The potent neuroprotective effects of neurotrophic factors, including brain-derived neurotrophic factor (BDNF), ciliary neurotrophic factor (CNTF), glial cell-line derived neurotrophic factor (GDNF) and nerve growth factor (NGF), suggest that they are good therapeutic candidates for neurodegenerative diseases. Glaucoma is a neurodegenerative disease of the eye that causes irreversible blindness. It is characterized by damage to the optic nerve, usually due to high intraocular pressure (IOP), and progressive degeneration of retinal neurons called retinal ganglion cells (RGCs). Current therapy for glaucoma focuses on reduction of IOP, but neuroprotection may also be beneficial. BDNF is a powerful neuroprotective agent especially for RGCs. Exogenous application of BDNF to the retina and increased BDNF expression in retinal neurons using viral vector systems are both effective in protecting RGCs from damage. Furthermore, induction of BDNF expression by agents such as valproic acid has also been beneficial in promoting RGC survival. In this review, we discuss the therapeutic potential of neurotrophic factors in retinal diseases and focus on the differential roles of glial and neuronal TrkB in neuroprotection. We also discuss the role of neurotrophic factors in neuroregeneration.

## 1. Introduction

Neurotrophic factors are a family of growth factors that play key roles in the development and survival of neurons. Neurotrophic factors generally include: the neurotrophin family (nerve growth factor (NGF), brain-derived neurotrophic factor (BDNF), neurotrophin-3 (NT-3) and neurotrophin-4/5 (NT-4/5)); the glial cell-line derived neurotrophic factor (GDNF) family (GDNF, neurturin (NRTN), artemin (ARTN), and persephin (PSPN)); and ciliary neurotrophic factor (CNTF), which is a member of the interleukin 6 (IL-6) family of cytokines. Each neurotrophin binds to one of the high affinity receptor tyrosine kinase (Trk family), with NGF binding to TrkA, BDNF and NT-4 binding to TrkB, and NT-3 binding to TrkC. They can also bind to the low-affinity neurotrophin receptor p75 (p75^NTR^) and induce programmed cell death, and these opposing effects of neurotrophins are important for regulation of developing neurons, including the retinal ganglion cells (RGCs) [1]. The potency of neurotrophic factors, particularly NGF, BDNF, CNTF and GDNF, in promoting neuronal survival has raised much hope for their therapeutic use in neurodegenerative diseases including Alzheimer’s disease, Huntington’s disease, amyotrophic lateral sclerosis (ALS), Parkinson’s disease and glaucoma [2,3,4].

Glaucoma is a neurodegenerative disease of the eye and is one of the major causes of irreversible blindness. It is estimated that by 2020, more than 80 million people will be affected worldwide, with at least 6 to 8 million of them becoming bilaterally blind [5]. Glaucoma is characterized by damage to the optic nerve, usually due to high intraocular pressure (IOP), and progressive degeneration of RGCs, which is a critical factor for vision loss. Currently, there is no cure for glaucoma and standard treatment focuses on reduction of IOP either by medication or surgery. However, some patients do not respond to this type of treatment. In addition, there is a subset of glaucoma known as normal tension glaucoma that is not associated with high IOP; however, many patients with normal tension glaucoma also benefit from lowering IOP [6]. Therefore, direct protection of RGCs from cell damage or death may be a novel therapeutic approach for glaucoma and could lead to a medical breakthrough; it may be particularly beneficial for those who do not respond to the treatments that are available at present.

In this review, we discuss the therapeutic potential of neurotrophic factors that are currently of clinical interest in retinal neurodegenerative diseases, particularly in glaucoma. We then focus on the differential roles of glial and neuronal TrkB in neuroprotection.

## 2. Neurotrophic Factors in the Retina

### 2.1. Nerve Growth Factor (NGF)

NGF belongs to the NGF family of neurotrophins and it can stimulate both TrkA and p75^NTR^. In the retina, TrkA is expressed in the RGCs and p75^NTR^ is mainly expressed in the Müller glia. Stimulation of these receptors in neurons can produce opposing effects, in which TrkA activation is associated with neuronal survival, whereas p75^NTR^ activation is implicated in neuronal apoptosis [7,8,9,10]. Indeed, in retinal degeneration models, TrkA activation exerts neuroprotective effects, but p75^NTR^ activation causes progressive RGC death [11]. Furthermore, selective TrkA agonists, but not NGF alone, induce protection of RGCs in an experimental rat model of high IOP and an optic nerve injury model [11,12,13]. These findings indicate that selective stimulation of TrkA is therapeutically beneficial in retinal degeneration and that therapeutic efficacy of NGF may be limited. However, we previously reported that RGC death in adult p75^NTR^ knockout (KO) mice following retinal ischaemic injury was almost identical to that in adult WT mice [14]. Furthermore, ocular application of NGF in a rat model of high IOP reduced the progressive loss of RGCs, and excitingly, it resulted in a progressive improvement of the inner retinal layer function and neural conduction in patients with advanced glaucoma [15,16]. This clinical study demonstrated long-lasting improvements in the visual field, optic nerve function, contrast sensitivity, and visual acuity; these findings are impressive, but the patient number was small [16]. Therefore, further studies are required to confirm the therapeutic efficacy of NGF eye drops in glaucoma patients.

### 2.2. Brain-Derived Neurotrophic Factor (BDNF)

BDNF was originally purified from the pig brain as a factor that promotes survival and neurite outgrowth of cultured embryonic chick sensory neurons [17]. BDNF belongs to the NGF family and is widely expressed throughout the central nervous system (CNS). BDNF exerts its pro-survival effects by binding to its receptor TrkB and activating signalling pathways involving the phosphatidylinositol 3-kinase (PI3K)/Akt, which leads to deactivation of proapoptotic targets, and the extracellular signal-regulated kinase (ERK), which results in phosphorylation of the cAMP response element binding protein (CREB) that induces transcription of various genes associated with neuronal survival [18,19,20]. It is a powerful neuroprotective agent, particularly for RGCs [21,22,23], and it is one of the key neurotrophic factors in glaucoma. It has been suggested that the blockade of axonal transport that leads to deficits in BDNF at the cell body may cause RGC death in glaucoma [24,25,26]. Live-cell imaging captured the dynamics of the axonal transport of BDNF in living RGCs and demonstrated that this activity was decreased before the death of RGCs [27]. Consistently, BDNF is reduced in the optic nerve head of glaucoma patients [28]. Interestingly, the BDNF levels in the serum of primary open-angle glaucoma patients and the tears of normal tension glaucoma patients are significantly lower than control subjects [29,30], suggesting that BDNF may be a biomarker for glaucoma.

While the effects of BDNF in protection of RGC are evident, therapeutic use of BDNF in CNS diseases has been greatly limited by the fact that BDNF does not cross the blood–brain barrier. Therefore, efforts have been made to target BDNF-TrkB signalling, particularly selective TrkB activation for promoting RGC survival. A bioactive compound, 7,8-dihydroxyflavone, with high-affinity at TrkB exerts neuroprotective effects in an animal model of Parkinson’s disease in vivo and in excitotoxic and oxidative stress-induced RGC death in vitro [31,32]. Other studies reported that antibodies that selectively activate TrkB enhanced RGC survival following acute and chronic models of glaucoma [33,34]. Furthermore, following optic nerve injury, a novel cell permeable phosphine-borane complex was reported to promote RGC survival through induction of retinal BDNF expression [35]. These findings indicate that selective activation of TrkB is a promising therapeutic target for retinal neurodegenerative diseases such as glaucoma. Alternative approaches to selective activation of TrkB include modulation of upstream or downstream regulators in this pathway, such as inhibition of the Shp2 phosphatase and GSK-3β activity [36,37,38].

### 2.3. Ciliary Neurotrophic Factor (CNTF)

CNTF belongs to the IL-6 family of cytokines and exerts robust neuroprotective effects on neurons. It binds to a receptor complex composed of the CNTF receptor-α (CNTFRα), gp130 and the leukemia inhibitory factor receptor (LIFR). Binding of CNTF to its receptor complex activates the Janus kinases/signal transducer and activator of transcription (JAK/STAT), mitogen-activated protein kinase (MAPK)/ERK, and PI3K/Akt signalling pathways [39]. Deletion of the *CNTF* gene in mice results in motor neuron degeneration and accelerated deterioration in inflammatory demyelinating disease [40,41]. On the other hand, mice lacking *CNTFRα* die shortly after birth [42]. In the retina, CNTF is expressed by various cells and particularly by Müller glia [43]. It is one of the most studied neurotrophic factors for protection of neurons in retinal diseases. It is highly potent against photoreceptor loss in several animal models of retinal disease, including retinitis pigmentosa [44,45,46,47,48] and against RGC death in models of glaucoma and ischaemic optic neuropathy [49,50,51,52,53]. The efficacy of CNTF in neuroprotection led to the idea of clinical use of CNTF. However, systemic administration of CNTF does not reach the CNS effectively [54]; therefore, direct application to the required site is desired. Excitingly, delivery of CNTF by encapsulated cell implants has been tested in clinical trials for treatment of retinitis pigmentosa and geographic atrophy, an advanced form of dry age-related macular degeneration (AMD) [55,56,57,58]. Application of this technology may also be beneficial for treatment of glaucoma.

In addition to neuroprotective effects, CNTF is capable of stimulating axonal regeneration [59]. Inflammation has been known to stimulate optic nerve regeneration [60] and it has been reported that inflammatory stimulation upregulates CNTF in retinal astrocytes, which in turn acts on RGCs to promote axonal regeneration [61]. Among the CNTF signalling pathways, the JAK/STAT pathway is negatively regulated by suppressor of cytokine signalling 3 (SOCS3) [62]. SOCS3 deletion in RGCs leads to robust axonal regeneration following optic nerve injury [63,64]. Strikingly, in an optic tract injury model, SOCS3 deletion in combination with various other factors associated with axonal regeneration including CNTF led to formation of functional synapses and recovery of visual function [65]. These are encouraging results for neural repair research and raise much hope for visual restoration as well as for functional recovery in other conditions associated with axon degeneration or injury in the CNS.

### 2.4. Glial Cell Line-Derived Neurotrophic Factor (GDNF)

GDNF belongs to the GDNF family and it is a distant member of the transforming growth factor-β (TGF-β) superfamily. It signals through by forming a multicomponent receptor complex composed of the glycosyl-phosphatidyl inositol (GPI)-anchored receptor GFRα1 and the transmembrane receptor tyrosine kinase RET [66,67]. GDNF was originally identified as a potent neurotrophic factor that promotes survival of midbrain dopaminergic neurons [68]. Although GDNF exerts powerful neuroprotective effects, mice that are deficient in GDNF displayed surprisingly minor deficits in the CNS and peripheral nervous system. However, these mice had profound deficits in the enteric nervous system and agenesis in the kidney, resulting in death soon after birth [69,70,71]. The neuroprotective effects of GDNF on various neurons suggested that it could be a good therapeutic candidate for neurodegenerative diseases including Parkinson’s disease and Alzheimer’s disease [72,73,74]. In the retina, GDNF stimulates survival of photoreceptors during retinal degeneration [75]. Recent studies have reported that long-term expression of GDNF in photoreceptors or retinal pigment epithelial cells using the tet/on inducible expression system slowed photoreceptor degeneration in rd10 mice, a mouse model of retinitis pigmentosa [76]. Interestingly, in the porcine retina, GFRα1 and RET are expressed in Müller glia, but not in photoreceptors [77], and application of GDNF to cultured mouse Müller glia upregulates BDNF, basic fibroblast growth factor (bFGF) and GDNF [78]. These findings suggest that GDNF may exert neuroprotective effects on photoreceptors indirectly via stimulation of glia. In addition, GDNF has been reported to protect RGCs following optic nerve transection [79,80] and following retinal ischaemia [81]. In a spontaneous glaucoma model, DBA/2J mice, intravitreal injection of microspheres containing GDNF significantly increased long-term RGC survival [82]. Microsphere-delivery of GDNF also stimulated survival of RGCs in an experimental glaucoma model [83]. Taken together, GDNF exerts neuroprotective effects directly and/or indirectly via glia, and GDNF holds strong therapeutic potential for retinal diseases especially retinitis pigmentosa and glaucoma.

Interestingly, GDNF upregulates the glutamate/aspartate transporter (GLAST) in Müller glia and this process is necessary for RGC protection following optic nerve transection [84]. We previously reported that deficiency in GLAST (GLAST KO mice) shows spontaneous RGC death and optic nerve degeneration without elevated IOP, similar to the pathological features observed in normal tension glaucoma [85]. GLAST is expressed in Müller glia and it is essential for keeping the extracellular glutamate concentration below a neurotoxic level. This process can prevent excitotoxic damage on surrounding retinal neurons [86]. Transporting glutamate into Müller glia is also important for synthesis of glutathione, a major cellular antioxidant in the retina. In GLAST KO mice, the glutathione level is decreased in the retina, particularly in Müller glia [85]. Reduction of the glutathione level is also observed in the blood of glaucoma patients [87,88]. Therefore, GLAST KO mice present with increased glutamate neurotoxicity and oxidative stress, and serve as a good model of normal tension glaucoma. Consequently, these mice have been providing extremely useful information on potential therapeutic strategies for normal tension glaucoma [89,90,91]. These findings indicate that GLAST plays critical roles in survival of injured RGCs and suggest that GLAST impairment may be involved in pathogenesis of glaucoma.

## 3. Neuronal BDNF-TrkB Signalling Stimulates the Dock3 Signalling Pathways and Promotes Optic Nerve Regeneration

**Figure 1:**
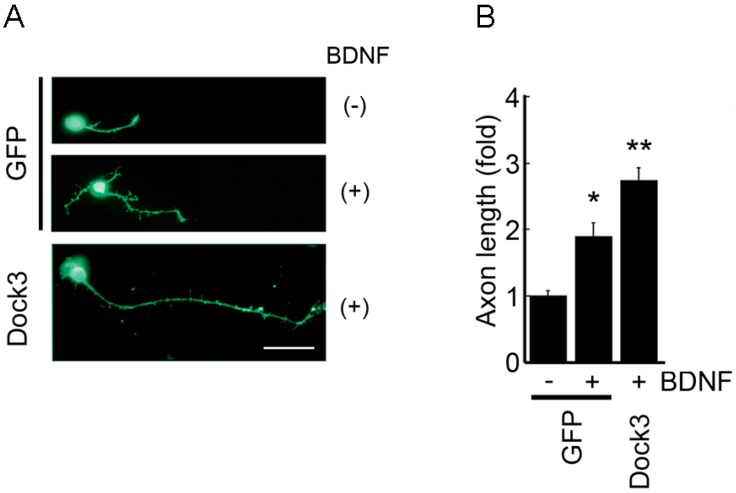
Dock3 enhances BDNF-mediated axonal elongation. (**A**) Retinal ganglion cells were cultured in the presence or absence of BDNF for 3 days. When Dock3 is overexpressed, the BDNF-induced neurite outgrowth is remarkably enhanced (Dock3). Scale bar, 20 μm; GFP, green fluorescent protein; (**B**) Quantification of (**A**). *n* = 50 for each experimental condition. Data are mean ± SEM of three independent experiments. * *p* < 0.05, ** *p* < 0.01. Modified from Namekata et al., *Proc. Natl. Acad. Sci. USA*, 2010, *107*, 7586–7591, Figure S3, ref. [97].

**Figure 2:**
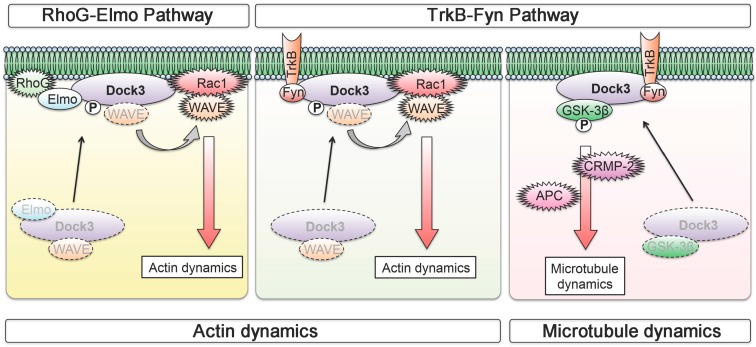
The Dock3 signalling pathways in optic nerve regeneration. Reproduced from Namekata et al., *Prog. Retin. Eye Res.* 2014, *43*, 1–16, ref. [95]. In the RhoG-Elmo pathway, Dock3 is recruited to the plasma membrane by the formation of a RhoG-Elmo-Dock3 complex, leading to translocation of WAVE and Rac1 activation. This signalling pathway stimulates actin dynamics (**left**); In the TrkB-Fyn pathway, Dock3 promotes both actin dynamics and microtubule dynamics. Recruitment of Dock3 to the plasma membrane by BDNF stimulation induces activation of Rac1/WAVE signalling and promotes actin dynamics (**middle**); GSK-3β is translocated to the plasma membrane by Dock3, where it is inactivated, leading to stimulation of microtubule dynamics via CRMP-2 and APC (**right**).

Dedicator of cytokinesis 3 (Dock3) is one of the atypical guanine exchange factors (GEFs) that regulate the activation of the Rho GTPase Rac1 [92]. Dock3 belongs to a family of Dock proteins and to date, 11 Dock proteins have been identified. These proteins play important roles in actin polymerization, migration and cell adhesion [93,94,95]. Dock3 is specifically expressed in the CNS and loss of Dock3 in mice leads to axonal degeneration associated with loss of integrity of axons [92,96]. These findings suggest that Dock3 plays a major role in maintaining cellular morphology and integrity of neurons for proper functioning. One of the activators of the Dock3 signalling is BDNF and in vitro, BDNF stimulates neurite outgrowth, in which overexpression of Dock3 enhances this effect [97] (Figure 1). Moreover, overexpression of Dock3 stimulates optic nerve regeneration in vivo *via* multiple pathways including GEF-dependent and GEF-independent pathways [97,98,99]. There are at least three pathways: the RhoG-Elmo pathway and, the WAVE-mediated and GSK-3β-mediated TrkB-Fyn pathways [95]. RhoG is one of the Rho GTPases and Elmo is an important effector of some of the Dock proteins including Dock3. RhoG directly interacts with Elmo in a GTP-dependent manner and forms a ternary complex with Dock proteins at the plasma membrane and induces Rac1 activation [99,100]. In the RhoG-Elmo pathway, actin dynamics are stimulated by the recruitment of Dock3 to the plasma membrane as a RhoG-Elmo-Dock3 complex, leading to translocation of WAVE. WAVE is an important downstream effector of Rac1 that is involved in axonal growth [101]. In the TrkB-Fyn pathways, one is associated with WAVE, which promotes actin dynamics, and the other involves GSK-3β, which stimulates microtubule dynamics. Stimulation of actin dynamics and microtubule dynamics has been shown to independently promote optic nerve regeneration [95,102]. GSK-3β plays a critical role in regulation of axonal growth during development [103], and its specific substrates, such as collapsin response mediator protein-2 (CRMP-2) and adenomatous polyposis coli (APC), have been identified as important mediators of axonal microtubule regulation [104,105,106]. BDNF-TrkB signalling recruits Dock3 to the plasma membrane, activates Rac1/WAVE signalling and promotes actin dynamics. Similarly, by directly binding to Dock3, GSK-3β is translocated to the plasma membrane, where it is inactivated, leading to stimulation of microtubule dynamics via modulation of CRMP-2 and APC. In sum, Dock3 stimulates multiple pathways and effectively promotes axonal regeneration (Figure 2). It is important to note here that whether BDNF can stimulate neuroregeneration in vivo is currently controversial [21,23,59,107,108]. Therefore, it is possible that the BDNF-TrkB pathway is merely one of the upstream signalling pathways for Dock3 and there may be multiple others that activate the Dock3 signalling pathway.

## 4. Glial BDNF-TrkB Signalling Upregulates Neurotrophic Factors and Promotes RGC Survival

**Figure 3:**
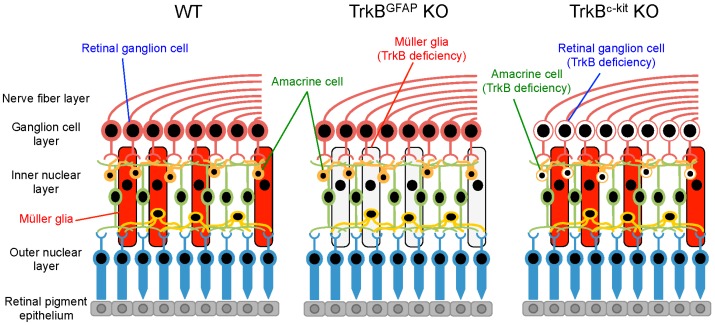
A schematic diagram of the retina with cell type-specific deletion of TrkB. In the TrkB^GFAP^ KO retina, TrkB from Müller glia are selectively deleted. In the TrkB^c−kit^ KO mice, TrkB from retinal ganglion cells and amacrine cells are selectively deleted. WT, wild-type.

**Figure 4:**
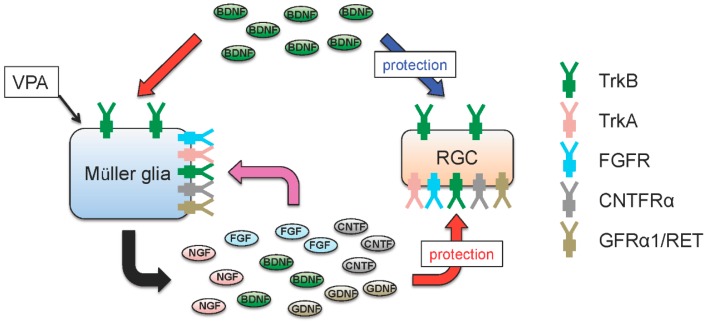
Neuroprotective effects of glial and neuronal TrkB signalling in the retina. BDNF exerts neuroprotective effects directly through TrkB expressed in retinal ganglion cells (RGCs) (blue arrow), and/or indirectly through TrkB expressed in Müller glia (red arrow). Stimulation of glial TrkB by BDNF upregulates various neurotrophic factors including BDNF, GDNF, FGF, CNTF and NGF (black arrows). These in turn increase neurotrophic factor production in an autocrine manner (pink arrow) and act on RGCs to promote survival in a paracrine manner (red arrow) [112,117]. In addition, stimulation of Müller glia by valproic acid (VPA) upregulates BDNF and NGF, which act on RGCs leading to neuroprotection.

It is well established that neuronal BDNF-TrkB signalling promotes neuronal cell survival by activation of prosurvival pathways. Interestingly, TrkB expression is relatively high in RGCs and Müller glia, but it is low in photoreceptors [109,110,111]. This suggests that the role of BDNF-TrkB survival signalling in RGCs and photoreceptors may be different. Indeed, we previously reported that treatment of Müller glia with BDNF in vitro upregulates CNTF and bFGF, which acts on photoreceptors and induce neuroprotective effects [112]. Subsequently, we demonstrated in vivo that stimulation of the BDNF-TrkB signalling in glial cells produces neuroprotective effects [113,114]. For this, Harada et al. generated conditional knockout mice targeting glial TrkB by crossing TrkB^flox/flox^ mice [115] with *GFAP*-Cre mice (Figure 3). Using these mice, it was shown that the degree of damage from glutamate neurotoxicity in mice whose *TrkB* is selectively deleted only from glial cells (TrkB^GFAP^ KO mice) and only from neurons (TrkB^c−kit^ KO mice) is similar. Moreover, photoreceptor degeneration induced by methylnitrosourea (MNU), an alkylating agent that causes photoreceptor apoptosis selectively in the retina [116], was accelerated in TrkB^GFAP^ KO mice. Furthermore, in an optic nerve injury model, RGC loss in TrkB^GFAP^ KO mice was more severe than in WT mice [114]. In cultured mouse Müller glia, treatment with BDNF upregulated BDNF, CNTF, GDNF and bFGF; these effects were absent in BDNF-treated cultured Müller glia prepared from TrkB^GFAP^ KO mice [113]. These findings suggest that glial BDNF-TrkB signalling promotes survival of neurons by supplying neurotrophic factors that stimulates neuroprotection. Taken together, these findings indicate that glial BDNF-TrkB signalling plays a neuroprotective role indirectly, by upregulating various neurotrophic factors which in turn stimulate prosurvival signalling in neurons (Figure 4).

Regeneration of neurons is severely limited in mammalian retinas, but in teleost fish, such as zebra fish, Müller glia respond to retinal injury in such a way that they undergo cell reprogramming and display characteristics of retinal stem cells [118]. Indeed, Müller glia are considered to be a type of radial glia that serve as progenitor cells capable of generating neural cells in the adult retina [119,120,121,122,123]. One of the key factors is reported to be Ascl1, which has been shown to reprogram mouse Müller glia to generate neurons [124,125]. Viral-mediated overexpression of *Ascl1* in cultured Müller glia induces retinal progenitor-specific genes and produces cells that express neuronal markers and neuronal activities [124], and transgenic expression of *Ascl1* in mouse Müller glia in vivo results in reprogramming of Müller glia into retinal neurons [125]. Interestingly, BDNF-TrkB signalling in Müller glia stimulates induction of rod photoreceptor markers and an RGC marker in proliferating Müller glia [113]. Therefore, Müller glial BDNF-TrkB signalling is not only involved in neuroprotection, but also in neurogenesis, indicating that TrkB in glia may be a good therapeutic target for neurodegenerative diseases.

## 5. Valproic Acid Upregulates Glial BDNF and NGF, Leading to Increased Neuronal Survival

Valproic acid (VPA) has been used clinically to treat epilepsy since the 1970s. It is a short-chain fatty acid and it is also used for treatment of mood disorders, migraines and neuropathic pain [126,127,128]. The diversity of its therapeutic use stems from its multiple pharmacological actions. Its antiepileptic features have been attributed to the stimulation of the brain γ-aminobutyric acid (GABA) transmission, and more recently, it was discovered that VPA is a class of histone deacetylase (HDAC) inhibitors [129,130]. HDACs are critical regulator of gene expression and HDAC inhibitors are clinically approved drugs for cancer treatment [131]. More recently, HDAC inhibitors are also explored for other conditions including neurological disorders and neurodegenerative diseases [132]. There is increasing evidence that VPA has neuroprotective properties and recent studies have shown some promising results in several animal models of neurodegenerative diseases including glaucoma, Alzheimer’s disease and Parkinson’s disease [91,133,134,135]. In an *N*-methyl-d-aspartate (NMDA)-induced neurotoxicity model, intravitreal injection of NMDA leads to acute RGC death [90,136]. Co-administration of VPA intravitreally prevents RGC death and visual impairment effectively [137]. One of the mechanisms associated with this neuroprotective effect is mediated via stimulation of the neuronal BDNF-TrkB pathway. We reported that VPA upregulates expressions of BDNF and NGF in Müller glia, and, using TrkB^c−kit^ KO mice, we demonstrated that the neuroprotective effects of VPA mediate activation of the neuronal TrkB signalling [137]. Although the NGF signalling pathway was not explored in this study, it is possible that NGF too has a role in VPA-mediated neuroprotection. Müller glia-derived neurotrophic factors play important roles in protection of photoreceptors and RGCs [112,113,138]. Therefore, compounds that mimic actions of such factors or those that induce their upregulation are of clinical interest. To this end, VPA may be a promising therapeutic candidate for retinal diseases including glaucoma.

## 6. Gene Therapy with Neurotrophic Factors

Neurotrophic factors stimulate neuroprotection and thus they have been considered a useful source for treatment of neurodegenerative diseases. To date, several clinical trials have been performed to treat neurodegenerative diseases by increasing supply of neurotrophic factors at the affected site. For example, NGF availability was increased in the forebrain of mild Alzheimer’s disease patients by autologous fibroblasts genetically modified to produce and secrete human NGF [139,140]; human GDNF was delivered via bilateral continuous intraputamenal infusion to Parkinson’s disease patients [141]; and CNTF was intrathecally delivered to ALS patients via an encapsulated genetically modified baby hamster kidney cells that release human CNTF [142]. These clinical data are not conclusive yet, but these are promising strategies that require continued development. In retinal neurodegenerative diseases, there has been increasing evidence to support the concept of neuroprotection as a therapeutic strategy and attempts have been made to enhance the level of neurotrophic factors in the retina to protect retinal neurons. Preclinical studies demonstrated that sustained expression of CNTF following adeno-associated virus (AAV)-mediated gene delivery results in remarkable protection of photoreceptors for a period of months in rodent models of retinitis pigmentosa [143,144,145,146]. Subsequently, the intraocular delivery of CNTF by the encapsulated cell implant was tested in clinical trials for retinal degenerative diseases [57,147,148], and conclusions from the therapeutic outcome are yet to be finalised. Alternative methods for intraocular delivery of neurotrophic factors may be use of stem cell transplantation. Stem cells that are genetically modified to produce and secrete neurotrophic factors have been successful in protecting retinal neurons. For example, transplantation of BDNF-secreting mesenchymal stem cells in the eye increased RGC survival in chronically hypertensive rats [149], and that of CNTF-secreting neural stem cells significantly reduced the loss of axotomized RGCs [150]. The safety and efficacy of this technology in the eye are currently tested in clinical trials [151].

Gene therapy has achieved some promising results for RGC protection in rodent models of glaucoma [152]. Virally mediated gene expression of BDNF in the retina promotes robust RGC survival in various experimental glaucoma models, including optic nerve transection, high IOP and intravitreal injection of NMDA [153,154,155,156]. Interestingly, BDNF gene delivery to Müller glia also prolonged RGC survival, indicating an important role of glia in neuroprotection [153]. Furthermore, overexpression of TrkB in RGCs can stimulate RGC survival following optic nerve transection [157]. These findings indicate that BDNF-TrkB signalling is a good therapeutic target for glaucoma. In addition, virally mediated overexpression of CNTF has also been reported to exert neuroprotective effects in experimental glaucoma models [52,158,159]. In summary, supplementation therapy using neurotrophic factors is an attractive strategy for neuroprotection in neurodegenerative diseases including glaucoma.

## 7. Conclusions

Neurotrophic factors play a major role in the survival of neurons, which is valuable in the treatment of neurodegenerative diseases. However, in practice, their clinical use is somewhat limited due to difficulties in delivery to the CNS and its poor pharmacokinetics profiles. Therefore, it is important to develop safe administration methods and determine the long-term effects of these proteins. In addition to encapsulated cell technology, stem cell transplantation technology is a promising strategy for delivering neurotrophic factors to required sites during retinal neurodegenerative diseases. Therefore, despite the difficulties, there is no doubt that neurotrophic factors are great therapeutic candidates for neurodegenerative diseases, and some of them that have overcome the problems have entered clinical trials. Unfortunately, neurodegenerative diseases like glaucoma are incurable with current medicine, but disease progression can be slowed or prevented. We believe that neuroprotection would be a novel therapeutic strategy for glaucoma and neuroprotection with neurotrophic factors may open up a new avenue in this field.
